# Increasing Occurrence of Marburg Virus Outbreaks in Africa: Risk Assessment for Public Health

**DOI:** 10.1111/1751-7915.70225

**Published:** 2025-09-02

**Authors:** Harald Brüssow

**Affiliations:** ^1^ KU Leuven Department of Biosystems, Laboratory of Gene Technology Leuven Belgium

**Keywords:** antivirals, control, ecology, epidemiology, filovirus, Marburg virus, vaccines, zoonosis

## Abstract

In this millennium, Marburgvirus (MARV) outbreaks with very high mortality but still small case numbers (< 400) were observed with increasing frequency in Africa. Ecologists identified Egyptian Rousettus bats (ERB) as viral reservoir species causing occasional zoonotic spillover events, mostly in humans intruding into their cave habitats as miners or tourists. So far only short human‐to‐human transmission chains have been documented. ERB can be experimentally infected with MARV but show no clinical signs. MARV transmission is inefficient among adult bats and occurs mostly between older juvenile ERB. WHO has modified infection control measures, requiring a high level of personal protective equipment when treating Marburgvirus disease (MVD) patients or burying the dead. If patients are quickly identified and isolated after symptom onset and contacts traced and also isolated, epidemics can be controlled. Researchers explored a number of antivirals against MARV in non‐human primate (NHP) MVD models. Compounds included galidesivir, an adenosine nucleoside analogue; favipiravir, a synthetic guanine base analog; remdesivir, an injectable; and obeldesivir, an oral prodrug which are intracellularly metabolised to an adenosine triphosphate nucleotide analog; small interfering RNA drugs that target short segments of the MARV nucleoprotein NP mRNA; and a human neutralising monoclonal antibody directed against MARV glycoprotein. All compounds mediated various levels of survival in challenged NHPs depending on dose and time of application. Various vaccine approaches (alphavirus replicons, adenovirus and vesicular stomatitis virus vectors, virus‐like particles, recombinant proteins, DNA vaccines) were explored in NHPs and conferred various degrees of protection against lethal MARV challenge. DNA vaccines were well tolerated in humans but showed only low immunogenicity. The African CDC has attributed an upper tier risk attribution to MVD when comparing 18 pathogens. For the moment, the short human MARV infection chains make large international outbreaks unlikely, but viral genome analysis in future outbreaks for transmission mutants is warranted.

## Graphical abstract

Bat and Marburg virus (Filovirus). Left: A *Rousettus aegyptiacus* bat; Center: a distribution map of *R. aegyptiacus* ; Right: EM of Marburg virus. (figure credits: Left Wikipedia Nilflughund Lithuanian Zoological Gardens, Center; Center: Wikipedia Nilflughund IUCN Red List of Threatened Species, right Wikipedia Marburg virus CDC Fred Murphy; J. Nakano).

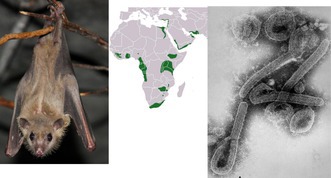

The human and economic impact of viral pandemics can be staggering as demonstrated by the recent COVID‐19 pandemic. A careful assessment of known and emerging viruses for their pandemic potential is critical to focus research and control measures on the most likely future pandemic threats. However, the WHO list of viruses that represent a potential threat to public health is long. The reduced financial support of the Trump Administration for The World Health Organization (WHO), the US Centers for Disease Control and Prevention (CDC), the US Agency for International Development (USAID) and infectious diseases programmes of The National Institutes of Health (NIH) necessitates a concentration of the available financial resources on research into the most likely candidates for future viral pandemics. A risk assessment for individual viruses is thus essential to develop a priority list.

In the current Lilliput contribution, a risk assessment is provided for Marburgvirus (MARV), a member of the Filovirus family to which also belongs the dreaded Ebolavirus. Ebolavirus and Marburgvirus represent two distinct genera in the Filoviridae family. While Ebolaviruses are represented by many species (e.g., Zaire, Reston, Sudan virus), the Marburgvirus genus consists of a single species (*Marburg marburgvirus*) with two representatives: Marburgvirus (MARV) and Ravn virus (RAVV), which differ in 20% of their full genome sequence (Kuhn et al. 2021). Ebolavirus and Marburgvirus differ more substantially in genome sequence (Figure 1) which necessitates a separate evaluation because knowledge on Ebolavirus cannot be directly translated to Marburgvirus. Both filovirus infections are characterised by very high case fatality rates (CFR) and require handling of the virus in biosafety level 4 laboratories. MARV was classified by WHO as a priority pathogen with a high risk of public health emergency of international concern (PHEIC). The present Lilliput contribution analyses the epidemiology and ecology of Marburgvirus infections, the control of outbreaks, candidate antivirals, and vaccines as a basis for an evidence‐based epidemic risk assessment for MARV.

**FIGURE 1:**
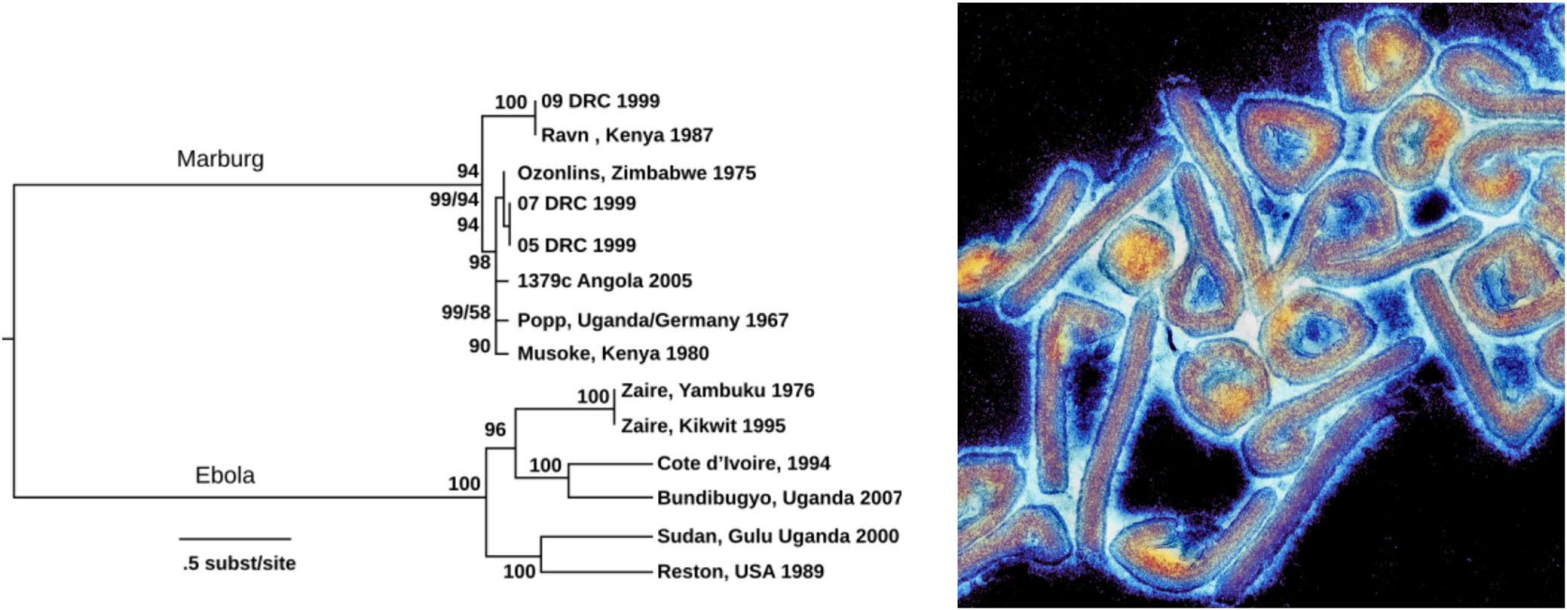
Marburg Virus, left phylogenetic tree, right electron microscopy of viral particles in false colour. Figure credit: Wikipedia Towner et al. and The University of Texas Medical Branch at Galveston.

## 1 Epidemiology of MARV Outbreaks

### 1.1 Laboratory Infections in Germany: 1967

MARV was isolated in 1967 during an outbreak among laboratory workers processing kidney cells from vervet monkeys for poliovirus production (Johnson 1982). Between August and September 1967, 30 cases of hemorrhagic fever occurred in the West German cities of Marburg and Frankfurt. Seven persons died; 5 secondary infections were observed in persons with contact to patients. The monkeys were traced to a single batch imported from Uganda. The velvet monkeys showed no signs of disease. However, green monkeys experimentally infected with MARV died within 12 days. An incubation time of 3 to 7 days was noted. Headache, fever, sore throat, myalgia, and diarrhoea were early clinical signs, followed by hepatitis, rash, pancreatitis, weight loss, asthenia, and psychological depression. Fatal cases developed melena, hematemesis, and bleedings from body orifices on day 6 of the disease. Death occurred by intravascular coagulation and shock before the tenth day of the disease. When laboratory work with these velvet monkeys was suspended, the outbreak ended.

The reservoir species for MARV could not be determined. Sera from several primate species in Africa were negative for MARV‐specific antibodies (Johnson 1982). Subsequently, only a few sporadic cases of human MARV disease (MVD) were reported from South Africa (Gear et al. 1975), Kenya, and a laboratory in Russia.

### 1.2 Democratic Republic of Congo (DRC) 1998

Between October 1998 and September 2000, 154 cases of MVD were reported in a mining district of DRC. CFR was 85%. Most cases were young male miners; secondary cases occurred in family members. Sequencing of the MARV genome revealed several distinct lineages, suggesting independent infection events, followed by short human‐to‐human infection chains. Clinical symptoms included bleeding, fever, fatigue, and vomiting. Death occurred 7 days after disease onset and was more prevalent in patients with conjunctivitis and hiccups. Health workers remembered similar cases since 1987. An ecological survey revealed bat colonies in the mining tunnels. The outbreak ended when the mines were flooded (Bausch et al. 2006). Virologists screened several hundred insectivorous bats (mainly *Rhinolophus eloquens* ) and fruit bats (mainly *Rousettus aegyptiacus* ). An estimated 10,000 bats inhabited the mine from which the epidemic started. About 3% of both *R. eloquens* and *R. aegyptiacus* tested positive for MARV RNA, and 10% and 20%, respectively, showed MARV‐specific IgG antibodies. Gene sequencing revealed several lineages of bat MARV within the mine that closely matched the different MARV sequences from human cases (Swanepoel et al. 2007). Household contacts from 73 MARV disease patients were screened for antibodies. Only 2 of the 237 household contacts were MARV seropositive. Both were unofficial gold miners and might also have contracted the infection in the mines (Borchert et al. 2006).

### 1.3 Angola 2005

Another MARV outbreak started in October of 2004 in northern Angola. The outbreak continued undiagnosed until March 2005 and ended in July 2005. Overall, 374 MARV cases causing 329 deaths were reported (CFR 88%). The MARV genomes were nearly identical, suggesting a single source for this outbreak. Médecins Sans Frontières (MSF) organised MARV isolation wards at the provincial hospital which reduced the nosocomial transmission of infections, but isolation was not well accepted by patients and their families. Oral fluid therapy showed only a small therapeutic effect (Jeffs et al. 2007). Standard biosafety procedures practiced in patient care and burials induced fear and anger in the population. MSF employed anthropologists and psychologists to alleviate fear but the high CFR undermined confidence. Contact tracing was essential to contain the epidemic (Roddy et al. 2007). MARV genomes showed only low sequence diversity (0.07%), suggesting introduction of the virus from a single source. Person‐to‐person transmission caused little accumulation of mutations. The Angolan MARV differed by about 7% from the viral strains isolated during the 1999 DRC outbreak (Towner et al. 2006).

### 1.4 Uganda 2007, 2012, and 2017

Uganda has seen repeated MARV outbreaks in 2007, 2012, and 2017. Four young men working in the Kitaka mine contracted MVD. Two cases were colleagues who cared for the index case A. They shared highly related MARVs. Index case B was epidemiologically unlinked and harboured a distinct MARV differing in 20% of its full genome sequence. It belonged to the distinct RAVN lineage of MARV. RAVN was first observed in 1987 in a patient who acquired the infection in a bat cave in Kenya. None of 83 contacts showed virological or serological evidence of MARV infection (Adjemian et al. 2011). The link between MVD, caves, and bats was underlined by two tourists who contracted MVD when visiting the Python bat cave in Uganda (Timen et al. 2009). The Kitaka mine was inhabited by at least 120,000 *R. aegyptiacus* and *Hipposiderus spec* bats. Virologists collected 600 *R. aegyptiacus* bats over two seasons: 5.1% of all and 10% of juvenile bats tested positive for MARV RNA. Furthermore, 2.4% of the bats showed MARV immunoglobulin G (IgG) antibodies, mostly in bats without viral RNA. RNA positive bats appeared healthy and showed only few infected cells in the liver and spleen. Five bat MARV genomes were sequenced: Two shared 99.3% sequence identity with MARV from index case A, and three showed up to 99.9% sequence identity with the virus from index case B. In contrast, only 1 out of 600 *Hipposiderus* bats was RNA positive for MARV, and none showed MARV‐specific IgG, identifying *R. aegyptiaca* as likely animal reservoir species (Towner et al. 2009).

In 2012, 26 MVD cases were identified; 15 were fatal. Three districts were affected and two infection chains were identified. Since all MARV genomes were nearly identical, they probably derived from a single spillover event. PCR tests revealed the highest blood viral load 1 week after symptom onset. In survivors, the virus became undetectable after 3 weeks. Specific IgM and IgG antibodies started to appear after 2 weeks. Surviving patients showed lower viral titers than fatal cases. Overall CFR was 58%. Anorexia, fatigue, vomiting, sore throat, and difficulty swallowing were observed. Bleeding was seen in half of the patients. Hiccups and anorexia were strongly associated with MVD diagnosis. Risk factors were contact with a case (odd ratio, OD: 33) and attending a funeral (OD: 5). No suspicious animal exposures were identified (Knust et al. 2015) In 2017, a farmer who had collected faeces as manure from a cave with a *Rousettus spec* roost died from MVD. Among 300 contacts, 3 family members were infected and two died (Nyakarahuka et al. 2019).

### 1.5 Ghana 2022

Three family members with MVD from Ghana shared 99.7% identity with MARV from a 2021 Guinea outbreak, traced back to a *R. aegyptiacus* isolate from 2017 (Bonney et al. 2023).

### 1.6 Tanzania 2023, 2025

A cluster of nine MVD cases was reported in Spring 2023 for Tanzania. The index case was a fisherman living near a cave inhabited by fruit bats. Six family members and among 212 close contacts, two healthcare workers contracted MVD. CFR was 67%; bleeding from orifices and vomiting blood were frequent observations in deadly cases (Mmbaga et al. 2024). In January 2025, ten MVD cases were reported at the border to Rwanda. All patients died. Control measures were implemented but none of the 90 contacts tested positive for MARV RNA, indicating low viral transmission (Venkatesan 2025).

### 1.7 Equatorial Guinea 2023

In Spring 2023, five MVD cases were reported in Equatorial Guinea. All were treated with supportive care and the antiviral drug remdesivir. Two patients died from encephalopathy, shock, and bleeding from the mouth and nares. The three survivors developed anorexia, epigastric pain, and rash. Survivors had a lower level of MARV RNA in the blood than the lethal cases (Fontana et al. 2024). An additional 39 MVD cases were identified, CRF was 90%. Five distinct transmission chains were detected, but all could be traced to a single virus introduction event. Secondary cases were family members, persons attending funerals of cases, and health care workers. Median time from symptom onset to hospitalisation and death was 4 and 8 days, respectively (Ngai et al. 2025). Surveys of caves identified MARV RNA‐positive *R. aegytiacus* bats that were related to MARVs from *R. aegytiacus* bats in Sierra Leone, while one MARV genome from a human case belonged to the Angola 2005 lineage (Makenov et al. 2023).

### 1.8 Rwanda 2024

In September 2024, Rwanda notified its first MARV outbreak with 66 cases and 15 deaths, mostly in health care workers (Uwishema 2024). Cases presented with fever, altered mental state, kidney and liver dysfunction. The patients developed seizures and blood oxygen levels decreased. When intubation was performed, massive bleeding from the oesophagus and lungs was observed. The index case reported contact with a fruit bat (Sibomana 2024). Experimental vaccines and monoclonal antibodies were provided and contact screening was done by CDC (Firew et al. 2025). Contact persons received a MARV vaccine candidate based on a modified chimpanzee adenovirus from the Sabin Vaccine Institute for “ring vaccination” (Callaway 2024). Comprehensive supportive care included mechanical ventilation which decreased the CFR to 23% (Grobusch et al. 2025; Nutt 2025). MARV genomes from early cases were highly related to each other and to a 2014 case in Uganda and a *R. aegytiacus* bat isolate from the Python cave in Uganda. Eight identical sequences were detected over the outbreak period, suggesting a single recent spillover event and very limited diversification during short human transmission chains (Butera et al. 2025).

### 1.9 Numerical Infection Parameters

For the Angola 2005 outbreak, epidemiologists deduced a reproduction number of R_0_ = 1.59. Without mitigation measures, MARV will be propagated with a doubling time of 12 days in a susceptible population. Serological data from DRC suggest an attack rate of 21%. The seroprevalence varied widely between African countries and the serological methodology used. For DRC, several studies reported a 2% seroprevalence. However, between 2% and 10% seropositivity was reported for African countries that never reported MVD, suggesting cross‐reaction with related but less virulent filoviruses. CFR varied widely per outbreak but could reach up to 90% without medical support. With more medical interventions as done in Rwanda in 2024, CFR could be decreased to 23%. The analysed data suggest that the clinical severity, death, and infectiousness are directly proportional to viral load. At the genomic level, MARV evolves three times slower than Ebolavirus (Cuomo‐Dannenburg et al. 2024).

## 2 Ecology of MARV Infections in Bats

### 2.1 Viral RNA and Antibodies

**FIGURE 2:**
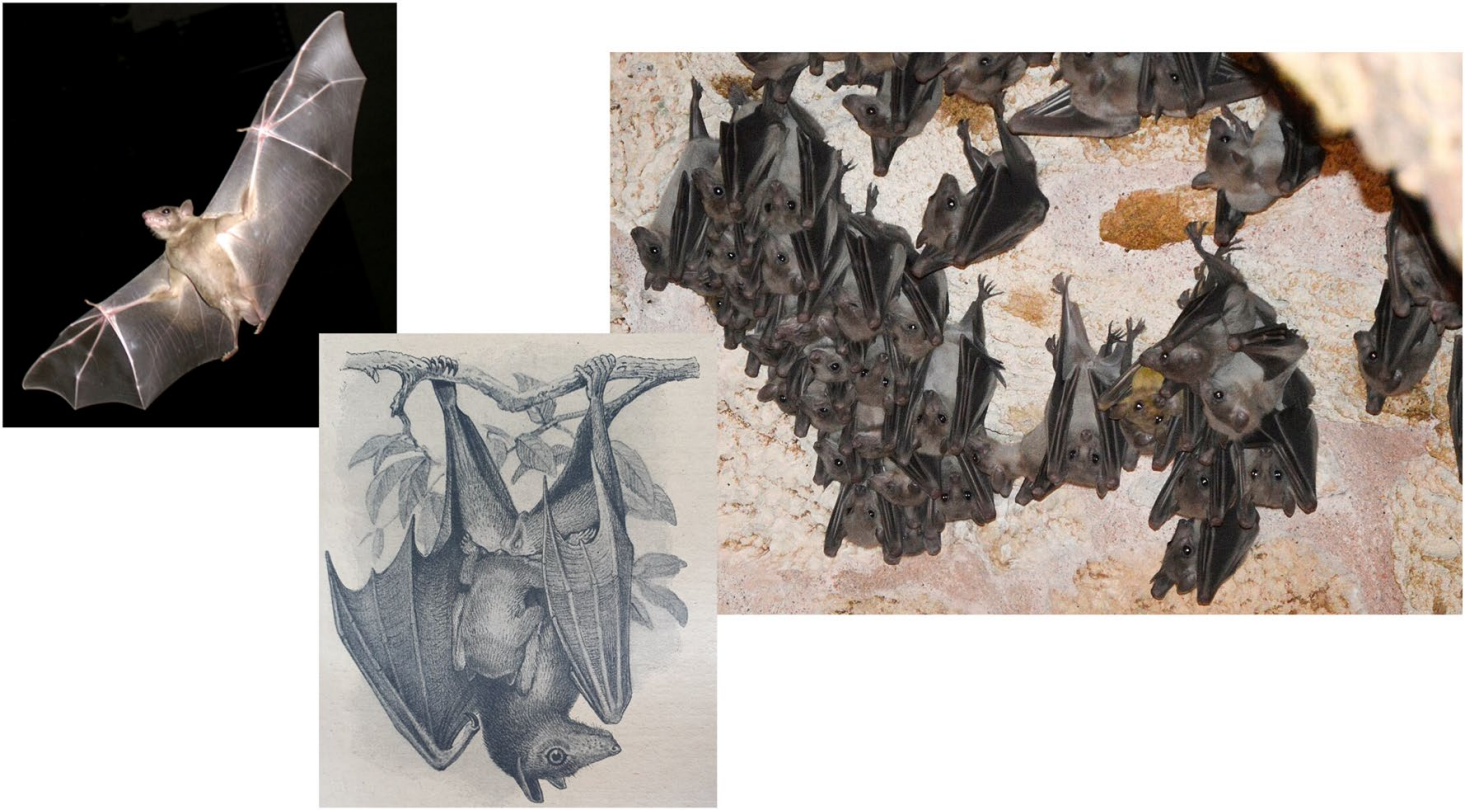
Egyptian Rousettus bat (ERB, *Rousettus aegytiacus*), left in flight, centre: Mother with young, right cave roost. Figure credit: Wikipedia PikiWiki Israel 11,327 and Natasha Haggard; Brehms Tierleben Säugetiere, erster Band Leipzig, Bibliographisches Institut, 1912.

Egyptian rousette bat (ERB), *Rousettus aegyptiacus* (Figure 2), belongs to the mammalian order of Chiroptera (the only mammal which developed powered flight), family Pteropodidae, and represents one of the few fruit bat species that use—due to their daytime resting in caves—echolocation for orientation. ERB are gregarious and social animals, living in densely packed roosts. ERB inhabits several scattered regions in Africa, the eastern Mediterranean, Arabia, and Iran along the Persian Gulf. ERB was shown to represent the natural host for both MARV and RAVV. When researchers analysed 1600 ERB from South Africa, between 0.9% (September) and 1.5% (April) of rectal samples tested positive for viral RNA. The reconstituted viral genome displayed high sequence identity with RAVV isolated in East Africa, suggesting a continent‐wide distribution of closely related MARV viruses among bats. Overall, 34% of ERB were seropositive, with the lowest seroprevalence of 5% in juveniles in April and the highest seroprevalence in September when 60% of juveniles were seropositive (Pawęska et al. 2020).

### 2.2 Geolocation of Foraging Range

About half of the African MARV outbreaks were linked with an encroachment of humans into caves where ERB rested during daytime. Ecologists equipped bats with GPS devices and tracked their foraging routes. Bats travelled a median of 60 km per night, intruded human homes and preyed on mango, papaya, avocado, or banana orchards (Amman et al. 2023).

### 2.3 Experimental Bat Infections

In experimental infections of ERB, RAVV induces viral titers of up to 10^4^ infectious virus per ml in blood and in oral secretions and lower titers in faeces. IgG seroconversion occurred after 3 weeks. The infection caused neither fever nor weight loss (Elbert et al. 2025). Comparable observations were reported for ERB after skin inoculation with MARV (Amman et al. 2015). The researchers dissected the animals and found viral loads throughout the body of the bats (with highest titers in spleen and liver). Disease symptoms were not observed and the infection was cleared after 4 weeks.

### 2.4 Viral Transmission

Since MARV and RAVV did not cause persistent viral infections in ERB, the constant MARV infection level in caves must be maintained by viral transmission from infected to naïve ERB. The high number of individuals and their physical proximity are obviously conducive to viral transmission. Naïve animals were co‐housed under various experimental conditions with experimentally MARV‐infected ERB. During the first 2 months of co‐housing, no naïve bats showed viremia or seroconversion (Paweska et al. 2015). However, after several months, 12% of the control bats showed viremia and 38% had seroconverted. The data showed that MARV can be transmitted, albeit with relatively low efficiency, between bats lacking ectoparasites (Schuh et al. 2017).

### 2.5 Arthropod Vectors?

Parasitologists have described Argasid ticks ( *Ornithodoros faini* ) as hematophageous ectoparasites of ERB. These ticks are vectors for the transmission of important veterinary viral diseases in Africa (African swine fever, bluetongue). Therefore, ecologists collected over 3000 of these ticks from bats, but none showed evidence of MARV RNA (Schuh et al. 2016). ERB also carry hematophagous fleas, namely *Thaumapsylla breviceps*. Five hundred fleas collected from ERB in a South African cave showed no MARV RNA by PCR. MARV RNA was detected in fleas that took a blood meal on viremic bats. However, neither seroconversion nor viremia was seen in control bats kept in close contact over a 40‐day observation period with MARV‐infected and flea‐infested bats (Pawęska et al. 2024).

### 2.6 Timing of Infections

The temporal analysis of the infection level in bats from different age groups provided insights into MARV transmission within bat colonies. ERB have two breeding seasons per year (May and November when juveniles are < 3 months of age). During that time period, MARV infection levels among juveniles were low (1.7% to 3%). In the rest of the year, when juveniles are older than 6 months, this rate was 10% to 16%. During this time period, juveniles represented the majority of all infected animals in the colony. This infection pattern is explained by the protection of young juveniles by their mothers (close contact and by maternal antibodies). In older juveniles, passive antibodies diminished and they exchanged the tight contact with their mothers for dense juvenile colonies in the lower parts of the cave, close to faecal deposits. This peculiar population dynamic provides two yearly pulses of virus‐susceptible bats. MARV can thus be maintained despite the low MARV transmissibility among adult bats. The majority of the documented MARV spillover events involving miners indeed occurred when the juvenile bats were 6 months old (Amman et al. 2012).

### 2.7 Interference in Dual Infections

ERB are also reservoir hosts for a Paramyxovirus (a relative of Nipah virus) and an orthonairobivirus (a relative of the Crimean‐Congo hemorrhagic fever virus), raising the possibility of positive and negative interference between concomitant virus infections. Viral ecologists observed that dual infection with Paramyxovirus and MARV reduced the oral MARV (peak MARV titers and duration of MARV excretion) compared to mono‐infected MARV ERB. In contrast, dual infection of ERB with both MARV and the orthonairobivirus increased MARV blood, oral, and rectal titres compared to mono‐infected bats. A few double‐infected bats became MARV super‐shedders (Schuh et al. 2025).

## 3 Outbreak Control Measures

The WHO revised its infection prevention and control (IPC) measures for Ebolavirus and Marburgvirus disease in 2023, based on newly acquired experience (WHO 2023; Willet et al. 2024). The recommendations included hand hygiene (alcohol rub or soap under running water instead of bleach and chlorine), personal protective equipment (PPE) to prevent contact with mucous membranes in the eyes, mouth, and nose (by face shields or surgical duckbill masks plus goggles or a particulate respirator in procedures creating aerosols from patients). PPE varies according to the degree of patient contact (home visit; transport to health facility, screening at hospital, patient triage, patient isolation, waste disposal at hospital and burial). If at patient screening a 1 m distance can be maintained (no touch approach), surgical scrubs and closed‐toe shoes are sufficient. If this distance cannot be respected at screening, gowns, medical masks, and one pair of gloves are recommended. At patient triage, scrubs and a coverall with face shield, two pairs of gloves, and rubber shoes are needed. WHO advised to disinfect the outer pair of gloves, remove the outer pair of gloves, disinfect the inner pair of gloves, and put on a new outer pair of gloves between patients. During patient care, an apron should complete the triage PPE. At the hospital, soiled linen should be incinerated, and surfaces should be cleaned by wiping and not spraying with disinfectants. The burial should be done in a respectful way; there is no need for disinfecting the body, but placing the dead in a body bag before burial is necessary. For the burial team, a respirator is recommended in addition to the full PPE since burials are high‐risk places for MARV transmission. WHO admits that these recommendations are based more on empirical plausibility than scientific evidence. An analysis of the 2005 MARV outbreak in Angola provided some indications for control measures (Ajelli and Merler 2012). Mean generation time for the virus was calculated to be 9 days with a latent period of 6 days, followed by an infectious period of 3 days. If timely performed in the first 3 to 10 days after symptom onset, case isolation is sufficient to contain a MARV outbreak. Infection rate could be reduced to 2 per 10,000 in a fully susceptible population by social distancing alone. The public health impact would thus be moderate, as also indicated by the relatively small case numbers in MARV outbreaks from Africa. In the 2023 MARV outbreak in Tanzania, an electronic Event‐Based Surveillance system was used relying on smartphones, thousands of information cards describing symptoms for the population, complemented by local radio announcements and information sessions in villages of the affected areas (Musyani et al. 2025). With this system, 176 signals were received; 27% met the MVD case definition, but only one case yielded MARV RNA. Two early cases in contact with the index case were missed because they showed unusual symptoms. These misses underline the importance of clinical symptom combinations for case identification. With data from the 2024 MVD outbreak in Rwanda using an MVD algorithm, fever, fatigue, nausea/vomiting, joint pain, and sore throat were identified by machine learning as key MVD predictors (Nsekuye et al. 2025).

## 4 Antivirals

### 4.1 Galidesivir

**FIGURE 3:**
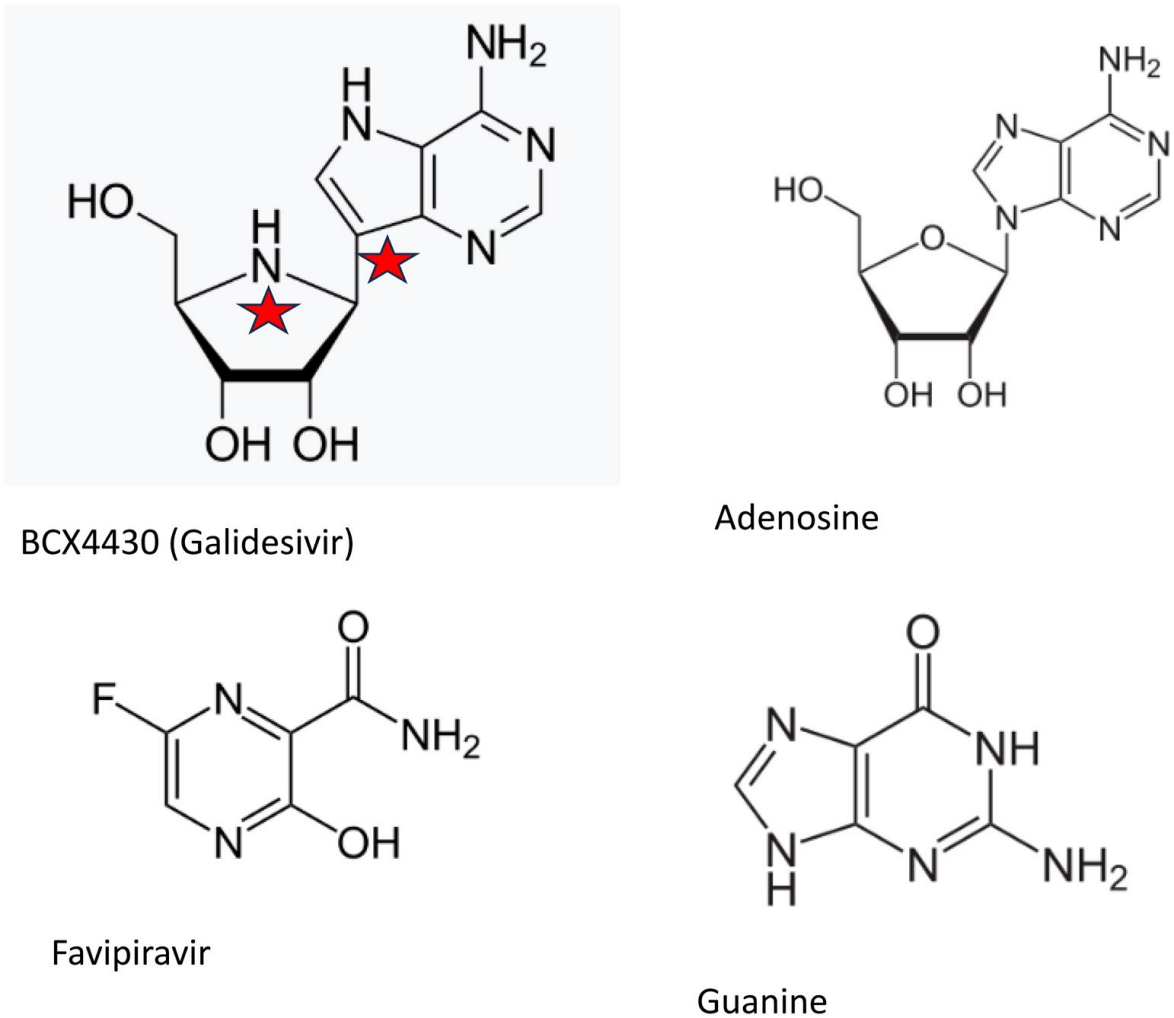
Antivirals: Nucleoside and base analogs. Top: BCX4430 (Galidesivir), an adenosine analog. Bottom: Favipiravir, a guanine analog. Formula credit: Wikipedia.

**FIGURE 4:**
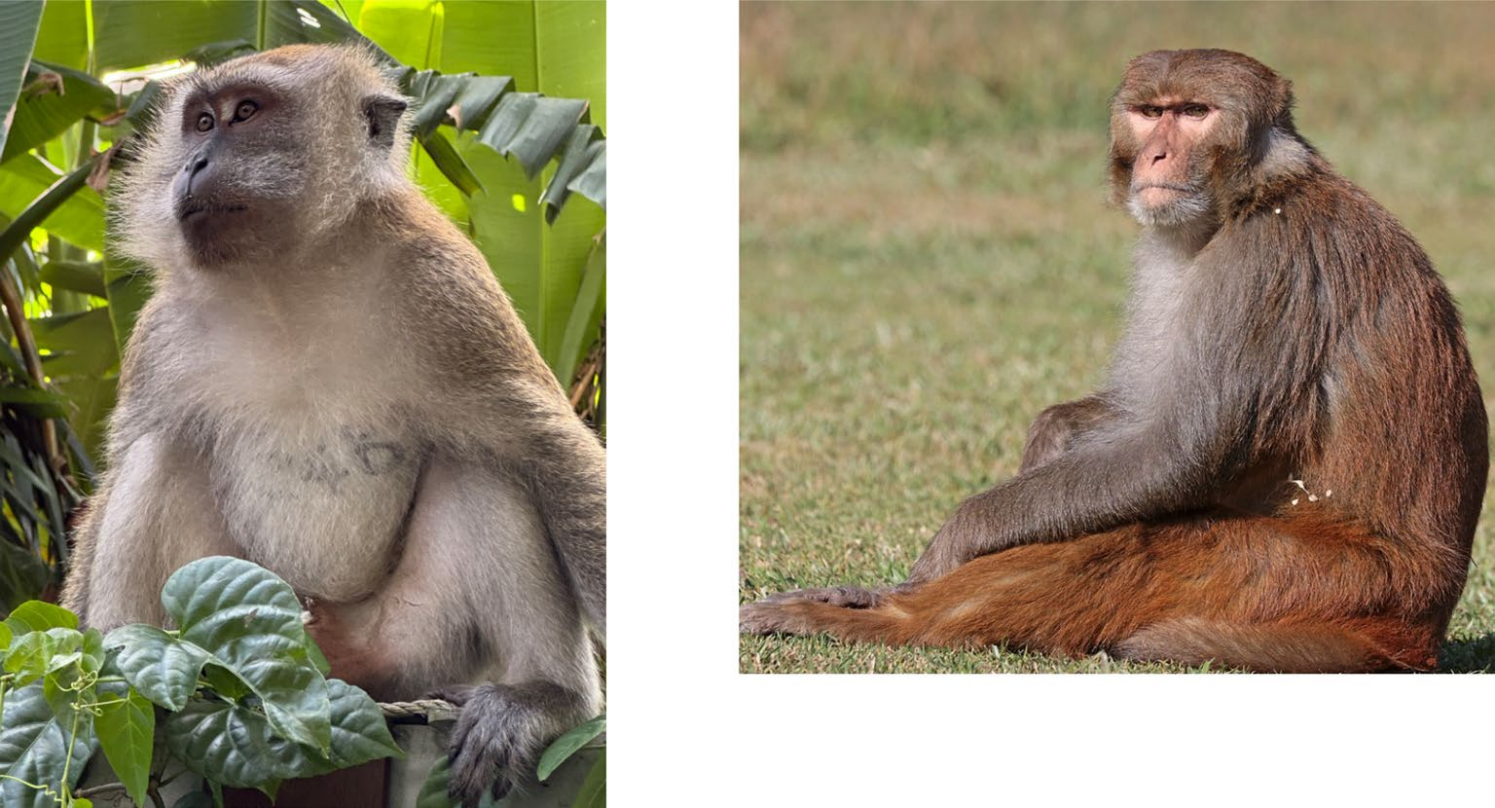
Cynomolgus and rhesus macaques, two non‐human primates that serve as gold standard animal models for MVD. Left: Cynomolgus macaque ( *Macaca fascicularis* ) and right: Rhesus macaque ( *Macaca mulatta* ). Figure credit: Wikipedia Charles Sharp.

US researchers synthesised BCX4430 (Galidesivir), a novel adenosine nucleoside analogue (Warren et al. 2014) (Figure 3). The prodrug is phosphorylated in cells and causes premature termination of transcription and replication of viral RNA, but has no effect on cellular RNA. It showed in cell culture a broad inhibition of several negative and positive strand RNA viruses, including MARV. Viral inhibition was observed at micromolar concentrations; cytotoxic effects occurred at 50‐fold higher concentrations. In cynomolgus macaques (Figure 4), a gold standard MVD NHP model which accurately reproduces human filovirus disease manifestations, BCX4430 was applied twice daily for 14 days at 15 mg per kg by intramuscular injection. The drug showed 100% protection against death when treatment was started 1 or 2 days after viral challenge, while all controls succumbed to disease. The drug reduced viral RNA production. Clinical chemistry showed that no liver injury or coagulopathy occurred in the treated animals.

### 4.2 Favipiravir

Favipiravir, a synthetic guanine base analog (Figure 3) and broad‐spectrum antiviral, was given to cynomolgus macaques intravenously shortly after challenge with MARV and treatment was continued for 2 weeks (Bixler et al. 2018). While all control animals succumbed to infection, 83% of the treated animals survived. Blood MARV RNA levels were reduced 10,000‐fold in the treated as compared to control animals and none of the treated survivors showed clinical or pathological evidence of MVD. None of their organs displayed viral antigen.

### 4.3 Remdesivir

**FIGURE 5:**
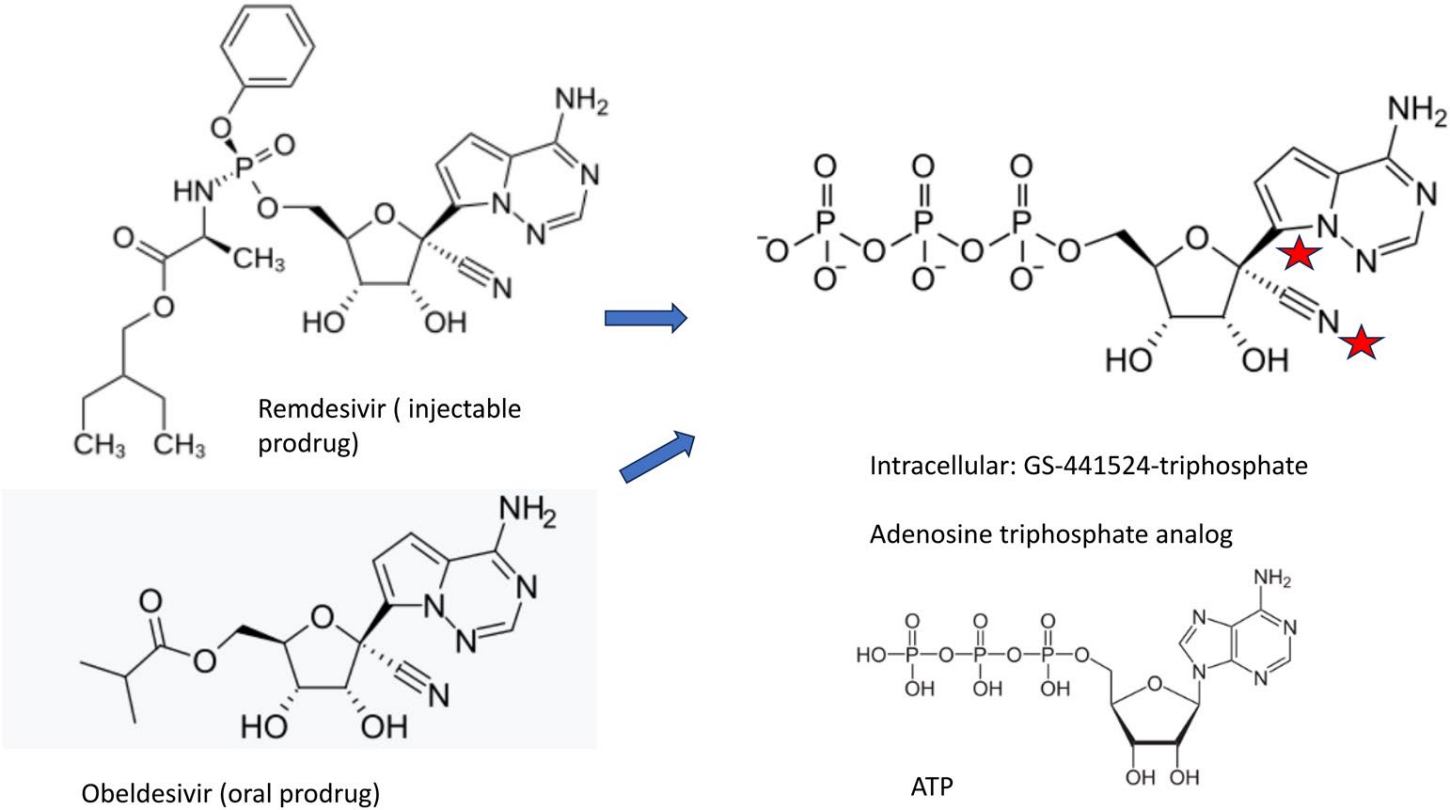
Antivirals: Injectable prodrug remdesivir and oral prodrug obeldesivir, which are intracellularly metabolised to the adenosine triphosphate analogue GS‐441524‐triphosphate. Formula credit: Wikipedia.

Subsequently, researchers explored remdesivir, an injectable prodrug for an adenosine triphosphate nucleotide analog (Figure 5) that inhibits viral transcription and replication by targeting the viral RNA‐dependent RNA polymerase. Groups of cynomolgus macaques were challenged with a lethal dose of MARV Angola strain and received either placebo (vehicle control) or remdesivir (Porter et al. 2020). All control animals died, whereas 83% of animals receiving a 10 mg/kg loading dose of remdesivir followed by a 5 mg/kg maintenance dose for 11 days survived, irrespective of whether treatment was started 4 or 5 days after viral challenge. Macaques receiving a 5 mg/kg remdesivir regimen, starting 5 days after viral challenge, showed a 50% survival rate. All drug‐treated animals showed a reduced and transient viremia. The surviving animals showed no or only moderate clinical signs, and only transient increases in liver transaminase, creatinine, and prothrombin time. Remdesivir is amenable to large‐scale manufacturing, has safety data for humans, but must be injected, which represents a disadvantage over orally applied nucleoside analogues.

### 4.4 Obeldesivir

Obeldesivir (ODV) (Figure 5) is an oral prodrug of a nucleoside ester that is metabolised in tissues to the same active triphosphate as remdesivir. US researchers challenged healthy adult cynomolgus macaques with a standard lethal dose of MARV Angola (Cross et al. 2025). Compared to a placebo control which succumbed to the infection after 7 days, 80% of monkeys survived the viral challenge when treated with 100 mg/kg ODV daily for 10 days, starting 1 day post infection. After transient low infectious titers in the blood, viremia was cleared in the surviving animals after 2 weeks. Viral RNA was also detected in lymph nodes from surviving ODV‐treated macaques, but infectious virus was not detected in any organ and no histopathological changes were observed. Transcriptome analysis showed that ODV treatment promoted adaptive immunity over excessive inflammation. However, these promising results must be extended to later drug application times than 1 day after infection since MDV patients are unlikely to present at this early stage at a health centre.

### 4.5 Inhibitory RNA Drugs

**FIGURE 6:**
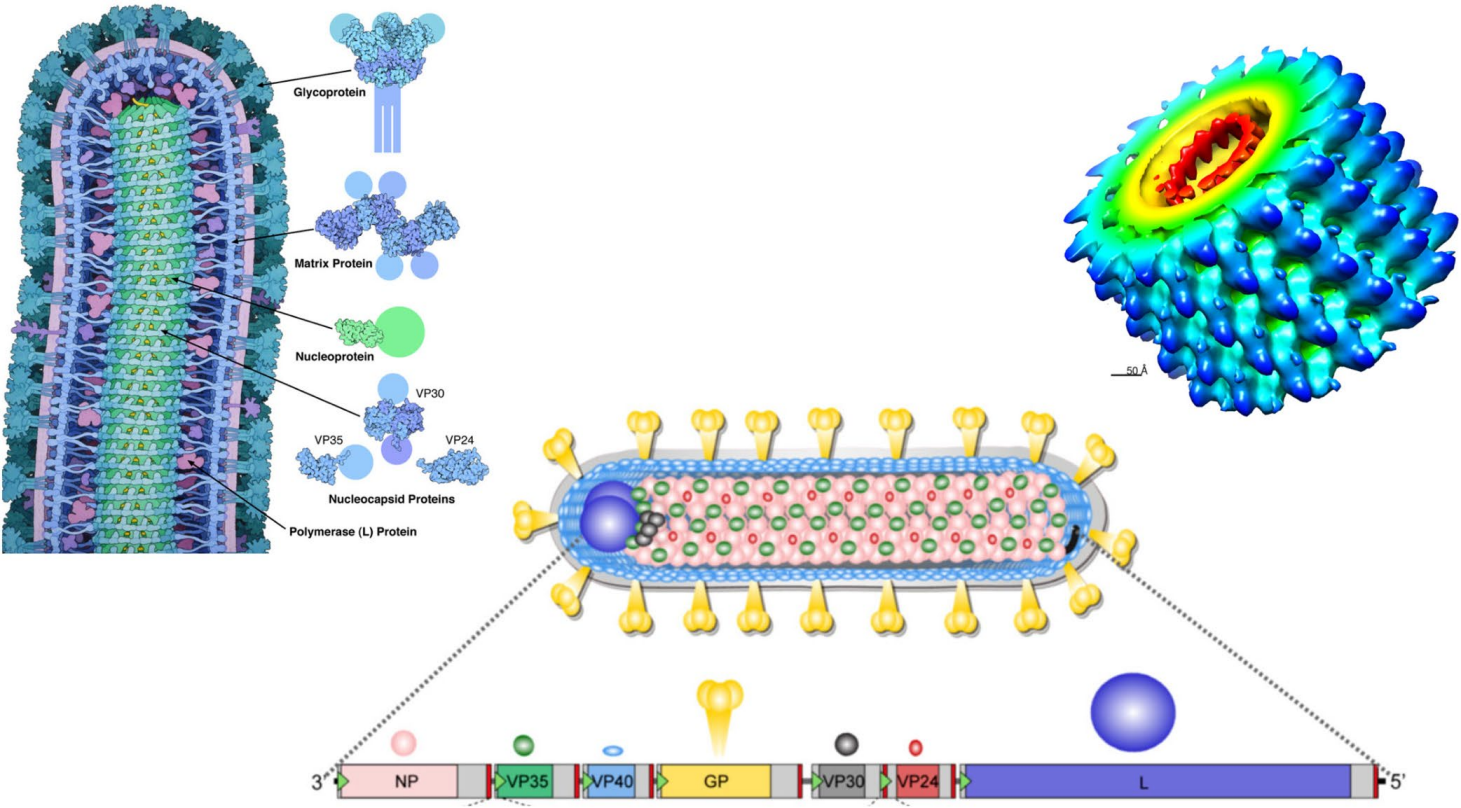
Marburg Virus structure. Left: Reconstruction of the end tip of a complete MARV virion. Center: Gene map of MARV genome with localization of the encoded proteins in the virion. Right: Reconstruction of a cut MARV nucleocapsid to show the localization of viral RNA (red) surrounded by nucleoprotein NP and nucleocapsid proteins. Figure credit: Wikipedia (David Goodsell RCSB PDB molecule of the month 178‐EbolaVirusProteins EbolaProteins.png; Kristina Brauburger et al. (2012); Marburg em1986.png).

**FIGURE 7:**
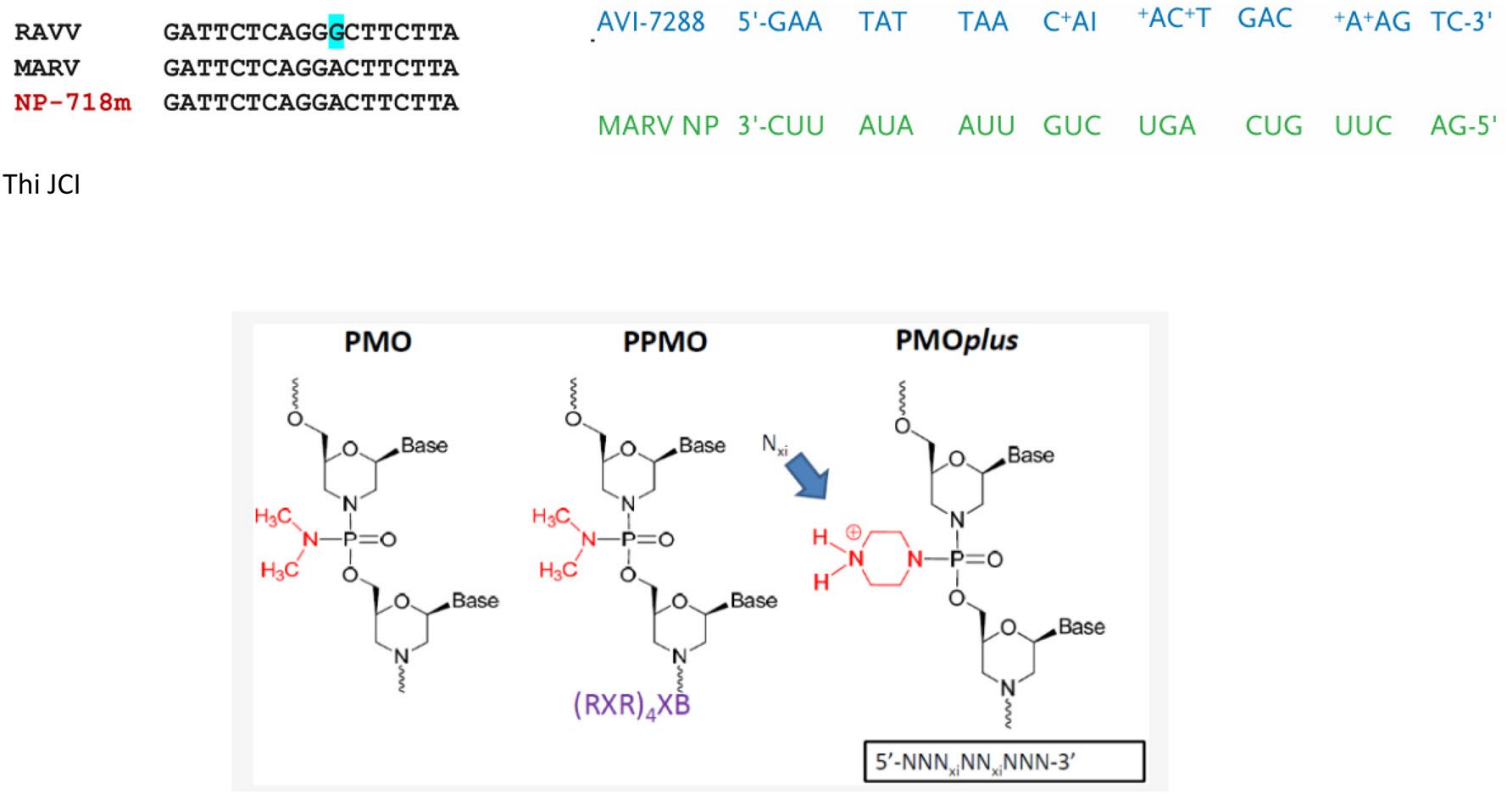
Antivirals: Inhibitory RNA oligomers complementary to MARV NP gene segments. Top left: the oligomers used by Thi et al. (2014, 2017); top right: the oligomers used by Heald et al. (2015). Bottom: The medicinal chemistry development of the PMOplus antisense RNA drug. Formula credit: Wikipedia Iversen et al. (2012).

US researchers targeted conserved sequence regions of MARV VP24 (secondary matrix protein), VP35 (polymerase cofactor), VP40 (matrix protein), NP (nucleoprotein), and Lpol (RNA‐dependent RNA polymerase) (Figure 6). Small interfering RNA (siRNAs) of 18 nucleotide length with 2′‐O‐methylation (to abrogate immune stimulation) were synthesised, annealed to form siRNA duplexes, and encapsulated into lipid nanoparticles (LNP) consisting of cholesterol and three amphipathic compounds forming spontaneously vesicles capable to include nucleic acids (Ursic‐Bedoya et al. 2014). Since RNA interference (RNAi) is a naturally occurring mechanism for the inhibition of gene expression, they expected antiviral effects against MARV. Significant inhibitory activity in cell culture infection was only observed with the NP‐specific siRNA. This was confirmed in guinea pigs challenged with MARV: 100% protection was only seen with NP‐718 m siRNA (Figure 7), another NP‐specific siRNA achieved only 40% and Lpol siRNA just 10% survival. The degree of protection correlated with the reduction in viremia. Only 60% survival was observed with NP‐718 m against RAVV, differing by only one nucleotide position in the target sequence. These were proof of principle trials because the siRNA was injected 1 h after viral challenge. Therefore, in a subsequent study, rhesus macaques were treated with NP‐718 m siRNA by intravenous bolus injection 1, 2, and 3 days after challenge with MARV Angola (Thi et al. 2014). All control animals succumbed to infection by day 9. In contrast, all treated animals survived until day 28, the end of the trial, even when treatment was delayed for 3 days after infection. Infectious virus and viral RNA were more than 1000‐fold reduced and not any longer detected after day 9. Viral RNA in the organs of treated animals was at the limit of detection. Liver aminotransferases showed only a transient increase and prothrombin time, which increased dramatically in control animals (indicating the coagulopathy characteristic for filovirus hemorrhagic fevers) remained at baseline in all treated animals. The treated animals showed no clinical signs of MVD. In a further follow‐up study, these researchers extended the delay of NP‐718 m treatment to 4 and 5 days after rhesus macaques were challenged with a lethal dose of MARV (Thi et al. 2017). Until that time, viremia with titers up to 10^8^ infectious virus per mL blood was observed in all animals. Viral titers decreased rapidly with the onset of treatment. Survival was 100% for MARV‐infected animals treated 4 days post infection (PI), but only 50% for those treated 5 days PI. Treated animals which succumbed to infection did not show mutations in the target region of the siRNA. The researchers also used NP‐718 m in macaques challenged with RAVV, which showed one nucleotide mismatch in the target region. A 100% protection rate was seen in animals treated with a 6‐day delay after infection, indicating that protection can be achieved with siRNA showing slight mismatches.

Another group of US researchers synthesised a 23‐mer antisense RNA (Figure 7) where the hydroxyl group of the phosphodiester bridge between ribose units was substituted at several places by a positively charged piperazine ring (PMOplus) (Figure 7) to prevent nuclease‐mediated RNA degradation (Heald et al. 2015). The sequence, modified by some inosine introductions, was complementary to a segment of the MARV NP mRNA. The antisense RNA associated with the NP mRNA and blocked its translation. When this antisense RNA was injected intravenously in cynomolgus macaques, survival of MARV challenged animals was dose dependent. Survival was 100% with a 20 mg per kg dose and decreased to 30% with a 3.75 mg per kg dose. Animals injected with placebo or antisense RNA targeting mRNA encoding MARV viral protein 24 or Ebolavirus proteins 24 and 35 all succumbed to infection. Delaying the drug injection for up to 4 days after viral challenge still conferred an 83% protection rate. The study was exceptional in using more than 130 macaques. The researchers also conducted a safety study in 40 human subjects with a 12 mg per kg dose and observed no significant adverse events. Pharmacokinetic analyses demonstrated a plasma half‐life for the antisense RNA of 4 h in both macaques and humans.

The serum viral load in surviving treated macaques was reduced 100‐fold as compared to control animals and all survivors cleared the viral RNA within 2 weeks. Viral load reduction was greater when the treatment started earlier after viral challenge. One mutation was seen in the viral RNA from treated macaques. It occurred in the NP encoding gene, but did not affect the binding of the anti‐sense RNA, excluding resistance development during treatment. Liver aminotransferases transiently increased in drug‐treated macaques but their liver showed no pathology nor viral antigens. Proinflammatory chemokines were significantly reduced in treated as compared to control animals (Warren et al. 2016).

### 4.6 Antibodies

A human survivor of a MARV infection showed a serum MARV neutralisation titre of 1:1000. US researchers screened the supernatants of transformed B cell lines from this survivor for binding to the glycoprotein (GP) of MARV. Positive hybridomas were tested for neutralisation of MARV; nine monoclonal antibodies (Mab) showed strong inhibition (Flyak et al. 2015) while Mabs binding MARV NP, VP35, or VP40 proteins lacked neutralising activities. Competitive binding assays of neutralising Mab showed that all bound to a single antigenic site of GP. Electron microscopy showed binding to the top of the GP near the binding site to the cellular receptor. This observation suggests that blocking of MARV GP binding to the host receptor is the principal mechanism of MARV neutralisation. The researchers screened for viral escape mutants, which all mapped to the receptor binding region. The GP recognising Mab MR191 also recognised the EBOV GP that lacked the glycan cap and mucin‐like domain. Upon intraperitoneal injection, several Mabs protected mice against disease when given concomitant with the viral challenge. These observations were confirmed by Chinese researchers with a different Mab (Zhang et al. 2024).

In a subsequent pilot study, rhesus macaques were treated with MR191‐N at day 4 and 7 after challenge with MARV (Mire et al. 2017). MR191‐N was produced in a tobacco (*Nicotiana benthamiana*) based rapid (1 month) antibody manufacturing platform using viral vectors. Plant‐specific glycosyltransferases were inhibited by RNAi, resulting in an afucosylated Mab which showed increased antibody‐dependent cellular cytotoxicity (ADCC) activity. At day 4, all macaques were viremic and had elevated body temperature. Treatment was associated with a rapid clearance of the plasma viral load. All three treated macaques survived and did not develop clinical symptoms, while controls died by day 9 PI. When 5 macaques were treated at day 5 and 8 with Mab MR191‐N, four animals survived. They all showed a rapid decrease in plasma viral load and no clinical symptoms. One treated animal did not survive: after initial viral load decrease, a viral rebound was seen that was, however, not associated with resistance development to Mab MR191‐N. All five macaques treated in the same way but challenged with RAVV survived and showed rapid viral load decrease in the blood. Similar results were reported by another research group working with a cocktail of three Mabs directed against three filoviruses including MARV (Brannan et al. 2019).

Production and purification of Mab for intravenous application can be time‐consuming and cost‐intensive. Canadian researchers explored a different form of passive immunity application. They used a muscle‐tropic Adeno‐associated virus (AAV) vector that expressed the MR191 antibody (Rghei et al. 2023). The vector expressed either the full‐length MR191 antibody, or variants with codon optimisation, or containing a host‐specific constant domain or containing two inflammation‐inhibiting oligonucleotides. Three months after intramuscular vector injection in guinea pigs, the animals were challenged with MARV. All treated animals survived the infection with minimal weight loss and no clinical signs. The inflammation‐inhibiting construct showed the highest serum antibody expression level. Protection was dose‐dependent: animals that received 5 × 10^12^ vector particles were fully protected while animals receiving ten‐fold less virus vector showed only a 30% survival rate.

### 4.7 Treatment Combination

Another treatment problem is the fact that in non‐human primate MDV models treatment could be delayed up to 5 days after viral challenge. Under field conditions, MDV patients will search for medical help later, mostly a few days after symptom onset. The question arose whether it is possible to extend the time window for a successful treatment by combining different treatment modes. This issue was explored by US researchers combining a half‐life‐extended version of the MARV MAb MR186 with the antiviral remdesivir (Cross et al. 2021). When treatment with only remdesivir or with only MR186 was delayed for 5 days, 80% and 100% of the macaques survived with moderate or mild clinical symptoms, respectively. When the treatment delay was extended to day 6 after viral challenge, all animals treated with MR186 or remdesivir alone succumbed to infection. In contrast, macaques treated with a combination of MR186 and remdesivir showed an 80% survival rate under these conditions. In contrast to single treatment modes, the combination treatment cleared the circulating virus rapidly. However, viral RNA was detected in the organs of surviving animals from the combination treatment group, albeit at 100‐fold reduced levels compared to single mode treatment.

## 5 Vaccines

Numerous vaccine constructs were developed against MARV. Here only those constructs are discussed that were tested in non‐human primate experiments. So far, MARV outbreaks are associated with low to moderate case numbers and relatively short duration; testing vaccines in human clinical trials is therefore difficult. The US Food and Drug Administration (FDA) has established the Animal Rule regulatory pathway for the licensing of MARV vaccines. According to that rule, a vaccine must demonstrate for licensing efficacy in a non‐human primate animal model, and an immune correlate of survival must be identified in animals and linked to human vaccine‐induced immune responses.

### 5.1 Alphavirus Vector Vaccines

An early vaccine candidate consisted of replicon‐helper systems derived from Venezuelan Equine Encephalitis (VEE) virus. The vaccine‐delivery system consists of a VEE replicon into which MARV genes are cloned, resulting in a self‐replicating RNA molecule that makes abundant MARV mRNA. This replicon RNA is transfected into eukaryotic cells along with two helper RNAs that express the VEE structural proteins (glycoproteins and nucleocapsid). The replicon RNA is thus packaged into VEE virus‐like particles by the VEE virus structural proteins, which are provided in trans. The resulting VEE replicon particles (VRPs) are infectious for one replication cycle but defective thereafter. Prescreening in a guinea pig MDV model showed that vectors expressing MARV GP or NP, but not those expressing MARV genes VP24, VP30 (a transcriptional activator), VP35, or VP40, suppressed viremia in challenged animals. Replicons expressing GP or GP plus NP protected cynomolgus macaques from viremia, while macaques receiving a vector with a control influenza gene died. Macaques receiving replicons expressing MARV NP mostly survived but developed viremia and became severely sick (Hevey et al. 1998).

### 5.2 Adenovirus Vector Vaccines

US researchers used a replication‐defective chimpanzee adenovirus expressing the MARV GP protein as a vaccine because this viral vector system had already demonstrated efficacy against Ebolavirus infections. A single‐shot intramuscular injection was chosen for practical reasons later in the field since the vaccine needs a cold chain for delivery. Cynomolgus macaques vaccinated with either 10^10^ or 10^9^ viral particles developed GP‐specific ELISA titers in excess of 3000. All vaccinated animals survived a lethal dose MARV challenge without developing viremia or clinical signs (Hunegnaw et al. 2022). With decreasing doses of 10^8^ to 10^6^ viral particles, decreasing ELISA antibody titers and decreasing survival rates were observed. An ELISA GP titre of 850 was still correlated with an 85% survival rate. Neutralising antibody titers were low and did not correlate with survival. Next, the researchers determined how quickly vaccine protection was observed. Already 1 week after vaccination, all macaques survived a viral challenge while high ELISA titers were only seen after 4 weeks of vaccination. Subsequently, the researchers determined the duration of vaccine protection by challenging macaques 6 or 12 months after a single‐shot vaccination. Survival rates were 100% and 75%, respectively, with ELISA titers of 870 and 450 at challenge.

The cAd3‐Marburgvirus (chimpanzee adenovirus type‐3 vaccine encoding a wild‐type MARV Angola glycoprotein) was then evaluated in a phase 1 trial with 40 US healthy adults (Hamer et al. 2023). The cAd3‐Marburgvirus vaccine is composed of a replication‐deficient cAd3 vector modified by an E1 region deletion and insertion of a codon‐optimised Marburgvirus Angola GP sequence. The vaccine was given as a single intramuscular dose with either 10^10^ or 10^11^ viral particles. The vaccine was safe and well tolerated. Most participants showed injection site pain, half reported malaise or headache, a third myalgia, and 1 subject showed fever for 1 day. MARV GP‐specific antibodies increased rapidly 100‐fold by ELISA and remained elevated over baseline for a year. Four weeks after vaccination, GP‐specific ELISA antibody mean titers were between 420 and 550, and after a year, titers were at 40. CD4 and CD8 T cells produced IFN‐γ, IL‐2, and TNF‐α in response to Marburgvirus GP peptide stimulation.

### 5.3 Vesicular Stomatitis Virus Vector Vaccine

Vesicular stomatitis virus (VSV, a Rhabdovirus) vectors that were already used in field trials for Ebolavirus ring vaccinations were also explored for MARV vaccination. To create the rVSV‐N4CT1‐MARV‐GP vector, the full‐length MARV‐Angola GP gene was cloned into a plasmid containing the full‐length VSV genome. MARV‐Angola GP was expressed at the first genomic position from the single 3′‐proximal promoter site to maximise GP antigen expression. VSV N gene was shifted from position 1 to 4 (N4) in the genome and the VSV G gene carried a cytoplasmic tail (CT1) truncation. Vaccine vectors were recovered from Vero cells following electroporation with the resulting plasmid construction along with VSV helper plasmids. To define the prophylactic window of this vaccine vector under outbreak conditions, cynomolgus macaques received 10^7^ virus vectors intramuscularly 7, 5, and 3 days before challenge with a lethal dose of MARV Angola strain. Survival was 100%, 80%, and 20%, respectively, demonstrating sound immunity when applied 1 week before exposure. Neither infectious MARV nor viral RNA was detected in survivors. Vaccinated survivors remained healthy and did not display clinical signs of MVD (Woolsey et al. 2022).

### 5.4 Virus‐Like Particles (VLP)

MARV GP, NP, and VP40 genes were inserted into a single baculovirus vector system for expression in insect cells. VLPs were recovered from the culture supernatants and purified. Cynomolgus macaques received three intramuscular injections of VLP over 3 months in saponin adjuvant. A strong ELISA antibody response against MARV GP was already seen after 2 weeks. The vaccinated macaques were challenged with a non‐lethal MARV dose by aerosol or subcutaneous injection. The VLP vaccinated animals showed no visible signs of disease, no gross pathology, and no viremia while animals receiving only the adjuvant became viremic and showed moderate disease signs (Dye et al. 2016).

### 5.5 Recombinant MARV GP Protein

Another group produced MARV GP from an Invitrogen expression vector in *Drosophila* insect cells. The recombinant protein was purified by immunoaffinity chromatography using an antigen‐specific monoclonal antibody, followed by size exclusion chromatography. After three intramuscular injections of 25 μg purified GP with adjuvant, cynomolgus macaques were challenged with an intramuscular lethal MRV Angola dose. The vaccine protected the animals against viremia, liver, and kidney damage. All vaccinated animals survived while all control animals receiving only the adjuvant succumbed to disease by day 9 (Lehrer et al. 2021).

### 5.6 DNA Vaccines

The MARV DNA vaccine consisted of a single, closed, circular plasmid DNA designed to express MARV GP. The MARV GP was cloned into the pWRG7077 eukaryotic expression vector downstream of the cytomegalovirus immediate‐early promoter. The GP insert was codon modified to optimise antigen expression in human cells. The plasmid is incapable of replication in human cells. DNA solution was intramuscularly injected with an insulin syringe within an electrode array. Injection of DNA was followed immediately by electrical stimulation.

Cynomolgus macaques were three times intramuscularly vaccinated at monthly intervals with 500 μg plasmid DNA. After the second injection, elevated ELISA and neutralising antibody titres against MARV pseudovirus were measured. MARV‐specific T cell responses were also observed in the interferon γ ELISpot test. Two months after vaccination, the animals were challenged with a lethal dose of MARV. Five of six vaccinated animals survived the challenge without demonstrating more than a transient viremia in one animal; no liver enzyme increases or clinical or pathological signs were observed. In contrast, animals vaccinated with an empty plasmid vector succumbed to infection at day 10 post challenge (Grant‐Klein et al. 2015).

Encouraged by these results, phase 1 clinical trials were initiated in humans. In Kampala/Uganda, 108 healthy subjects were allotted to four groups. The DNA vaccine groups received MARV GP gene, EboV GP gene, or a cocktail of MARV and EboV GP genes; a fourth group received placebo. Vaccines were applied by three intramuscular needle‐free injections, spaced 3 weeks apart (Kibuuka et al. 2015). All vaccines were well tolerated. No significant difference in reactogenicity was seen when compared to placebo recipients. Glycoprotein‐specific ELISA antibodies peaked 4 weeks after the third injection but were weak. In the MARV DNA vaccine group, 31% of the vaccinees showed a response, but the mean geometric titre was not significantly higher than that seen in the placebo group. T‐cell responses, as assessed by interferon γ ELISpot tests, were only seen in 3% of the MARV DNA vaccine recipients. A pilot study in 10 US volunteers confirmed these observations (Sarwar et al. 2015). MARV GP‐specific ELISA antibodies were measured in 80% of the subjects, but titers were low; they increased after a fourth injection but then decreased again to baseline levels. Neutralising antibodies were not detected. GP‐specific CD4+ T‐cell responses assessed by intracellular cytokine staining were detected in 30% of subjects.

## 6 Conclusions: Risk Assessment

Epidemic risk assessment is a complex task since many factors must be considered. The degree of pathogen circulation (which is influenced by climate change and human mobility), the transmissibility of the pathogen, the susceptibility of the population, mortality rates, severe infection sequels, and the economic and social impact of an epidemic enter into the calculation. Risk attribution decreases when countermeasures exist such as anti‐microbial drugs, vaccines, and efficient epidemic containment measures. Based on these considerations, a risk assessment has been elaborated for 18 priority pathogens by the African CDC. In a two‐dimensional scattergram, plotting epidemiological potential against disease severity, the top ranking risk for Africa was associated with Ebolavirus. MARV occupies an upper tier position: only COVID‐19, cholera, and yellow fever got higher rankings. MARV risk was rated as great as that posed by Lassa fever, Neisseria meningitis, and Crimean‐Congo hemorrhagic fever (Risk Ranking and Prioritization of Epidemic‐Prone Diseases—Africa CDC).

Let's consider the epidemic/pandemic risk of MVD on the basis of the observations summarised in this Lilliput review. The entire world population is susceptible to MARV infection as demonstrated by the original MVD outbreak in Germany and by tourists from different continents contracting MVD in Africa. The case fatality rate is one of the highest of any infectious diseases. The potential for social disruption is also high as demonstrated by the panic reaction of the African population in outbreak areas.

A different picture emerges when considering the transmissibility of MARV. MVD is a zoonotic disease with a known viral reservoir in *R. aegytiacus* fruit bats. As long as no sustained human‐to‐human infection chains are documented, the geographical risk area for MVD is thus restricted to the geographical range of *R. aegytiacus*, which is Africa, the Eastern Mediterranean and the Near East. However, MVD cases have not been reported outside of Africa possibly because *R. aegytiacus* bats do not carry MARV outside of Africa.

Models using the distribution of MARV infected bats restrict the risk area to Equatorial and West Africa, putting 105 million people at risk (Pigott et al. 2015). Indeed, ERB from Sierra Leone/West Africa showed a 2.5% prevalence of MARV carriage. These viruses were genetically related to viral strains associated with the 2005 outbreak in Angola (Amman et al. 2020). However, no MVD cases were documented over the last few decades in Sierra Leone. MARV carriage in ERB might thus be a necessary but not sufficient condition for zoonotic MARV. This observation suggests that additional risk factors are needed for MARV transmission to humans, such as mining in ERB inhabited caves. Amman et al. (2020) therefore warned against a prospective culling of ERB in Sierra Leone, since a theoretical infection risk reduction might be well offset by the ecological service of fruit bats for forest regeneration by dispersing seeds and facilitating pollination.

Models of MARV risk assessment have been developed based on the distribution of human MVD cases which indicate that 75 million people from 19 African countries, mostly surrounding DRC, are living in high‐risk MARV areas (Pigott et al. 2015). While the pace of MVD outbreaks is increasing in Africa (as has been observed for viral epidemics in general), MVD outbreaks still affect relatively small numbers of subjects. This seems to indicate that MARV has not (yet) achieved sustained infection chains in humans. Transmission currently needs close contact with MVD cases as occurring in households, medical care, and burials. As long as MARV does not mutate to increased transmissibility in humans, the epidemic potential in Africa will be limited to small outbreaks and the danger of a pandemic outside of Africa is low. The selection pressure for MARV to mutate to better transmission among humans might not be so high as it could compromise transmission in their animal host (another reason against bat culling since such measures could force MARV into humans). It will be wise to conduct sequence analysis of MARV isolates across future MARV outbreaks to assess the risk of such mutations. The large Ebolavirus epidemic in West Africa between December 2013 and April 2016, associated with 28,000 cases and causing 11,000 deaths (WHO Ebola Response Team et al., 2016), should serve as a warning. It was traced to a single zoonotic spillover event (Baize et al. 2014) followed by extensive human‐to‐human transmissions (Gire et al. 2014). This epidemic justifies the high‐risk ranking for Ebolavirus in West and Equatorial Africa, while for MARV the epidemic/pandemic risk is (at least for the moment) substantially lower.

However, viral‐host interaction is a dynamic scene. Researchers observed that MARV‐infected ERB cells upregulate canonical antiviral genes with minimal proinflammatory gene induction, suggesting a mechanism for disease tolerance in the reservoir species (Guito et al. 2021). However, when ERB were treated with dexamethasone, an anti‐inflammatory agent, increased MARV replication and higher oral and rectal MARV shedding were observed (Guito et al. 2024). The researchers suspected that stressors like food scarcity and habitat disruption could have the same effect and represent important co‐factors for spillover events as had been previously demonstrated for Hendra virus spillover infections from *Pteropus* fruit bats in Australia (Becker et al. 2023; Eby et al. 2023).

Finally, in the African CDC risk assessment, Ebolavirus infections have a better preparedness score than MARV infections. While this is true, preclinical trials in nonhuman primate MVD models showed promising candidates for several classes of antivirals and vaccines against MARV. In addition, empirical guidelines have been developed for non‐pharmaceutical interventions capable of containing MARV outbreaks if followed at the beginning of an outbreak.

Overall, this evaluation allows a less dramatic ranking for the risk of large MVD epidemics in Africa or internationally. However, I have not mentioned a political risk factor favouring epidemic spread in the future. The current US administration dramatically reduced the financing of epidemic support in developing countries, of vaccine research and of fundamental research in infectious diseases. In view of the large contribution of US science to this field in the past, we will be less well prepared when confronting the next epidemic without US scientific support, increasing the risk of such epidemics running out of control.

## Author Contributions

**Harald Brüssow:** conceptualization, investigation, writing – original draft.

## Conflicts of Interest

The author declares no conflicts of interest.

## Data Availability

The author has nothing to report.
